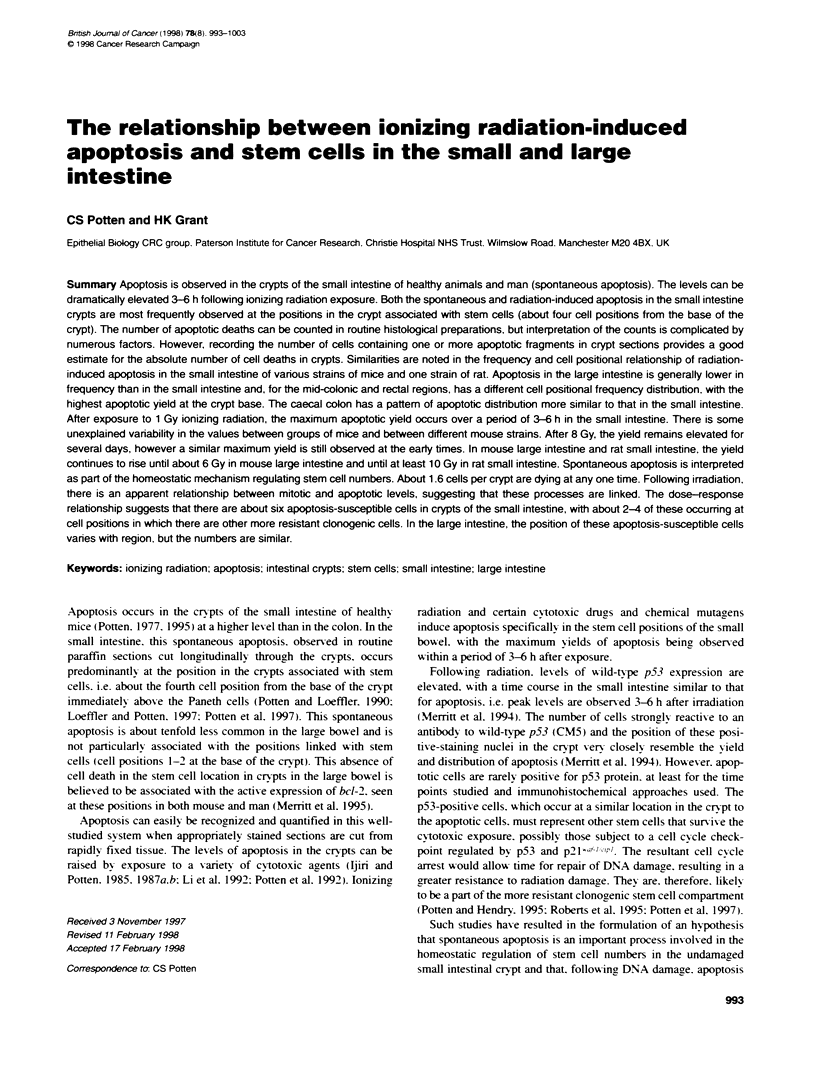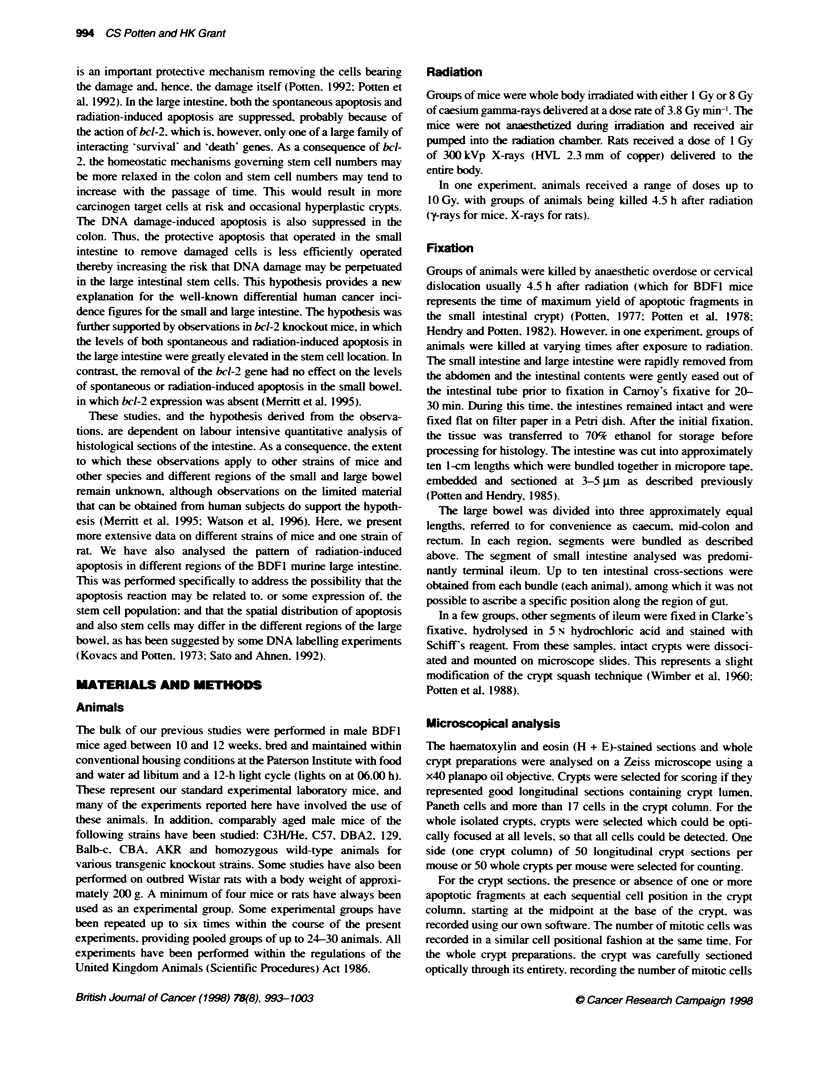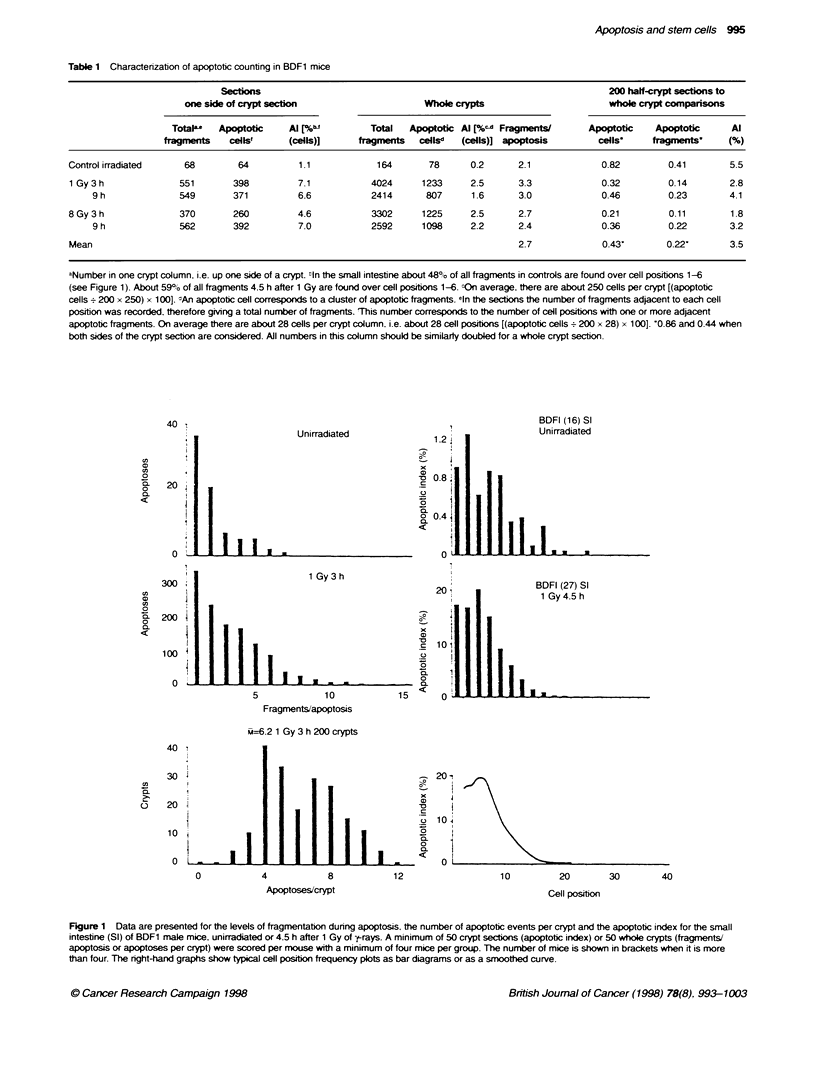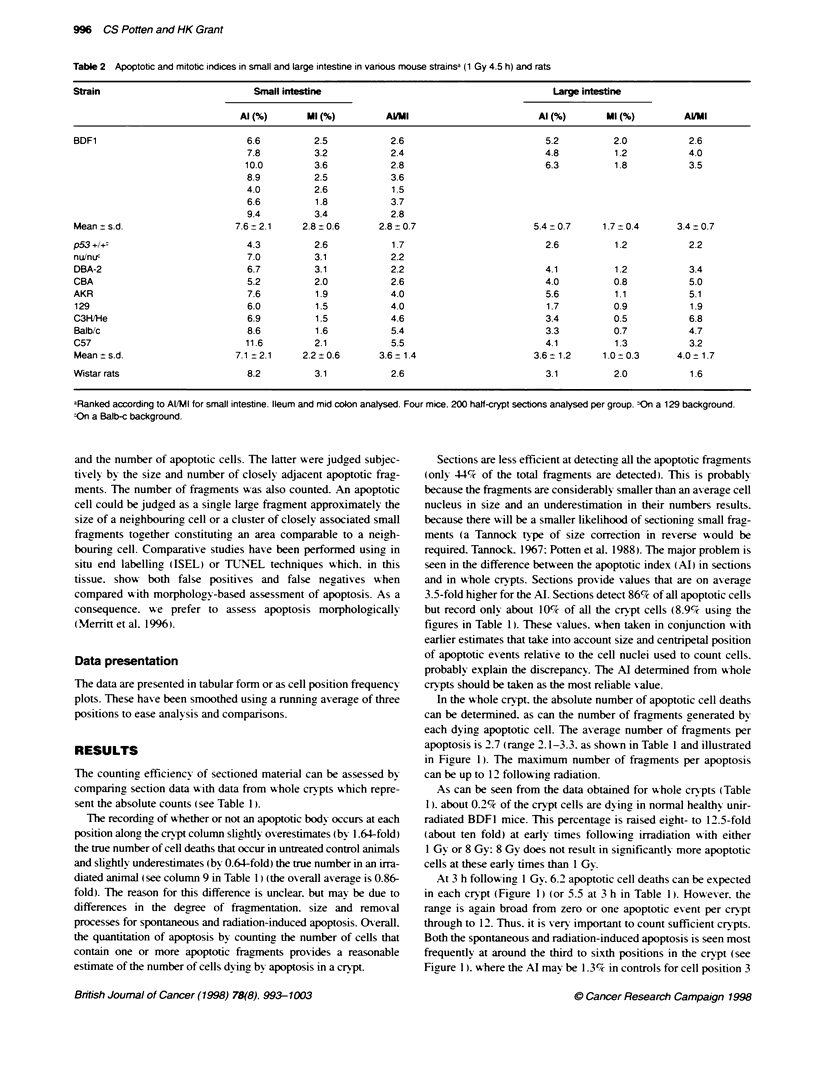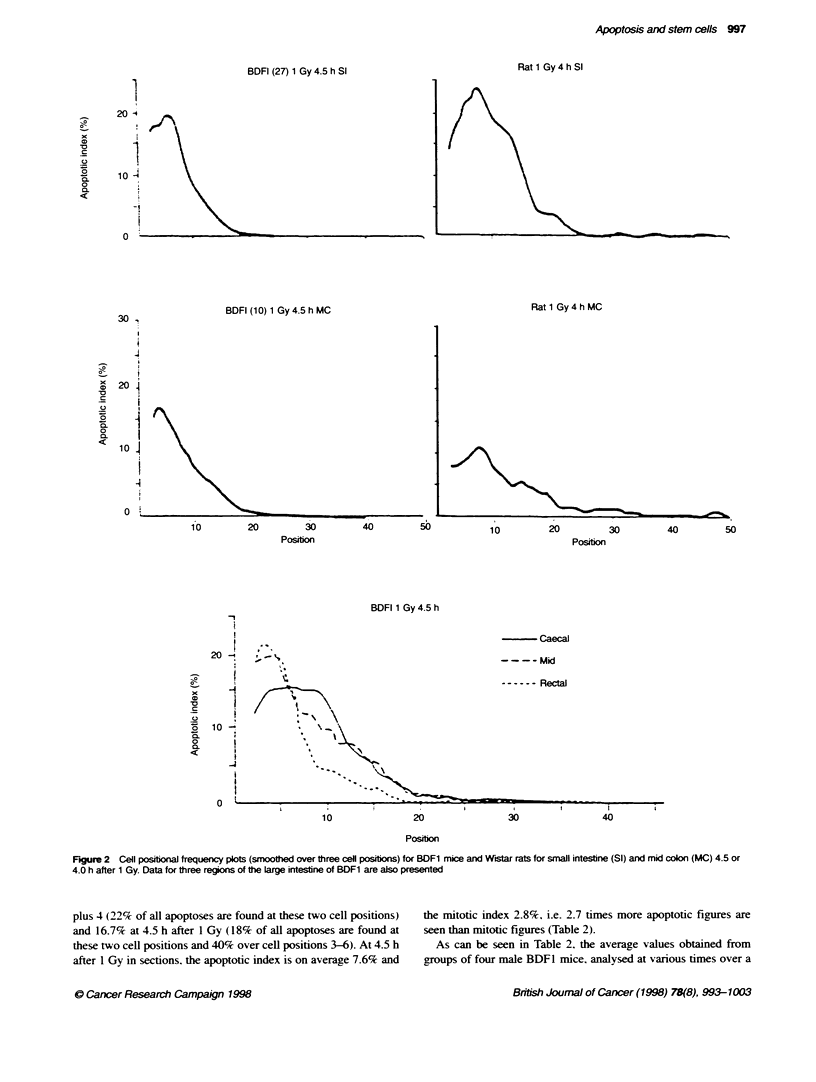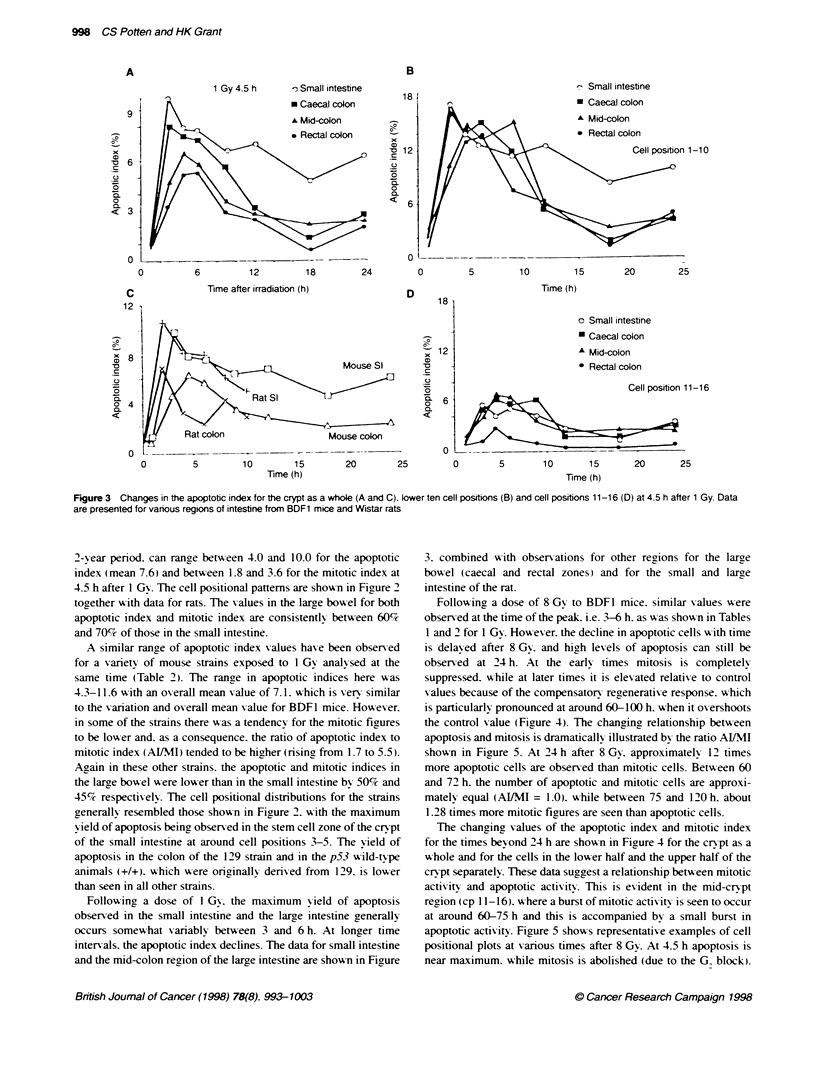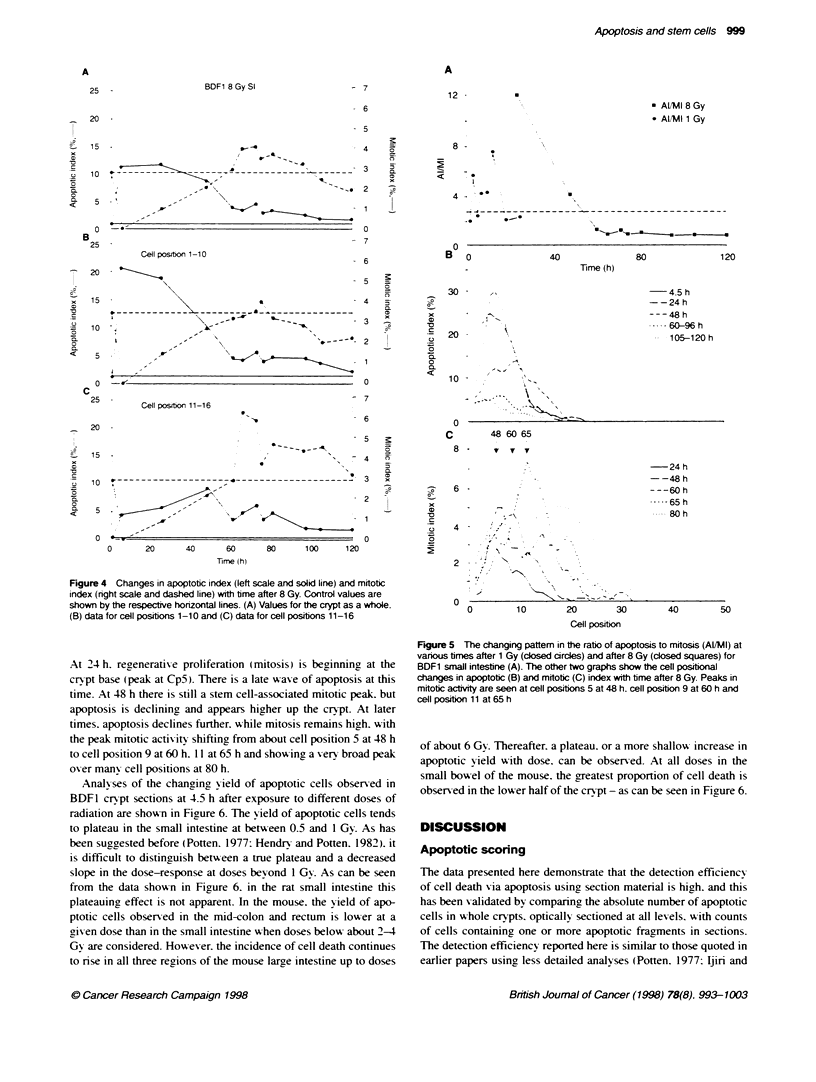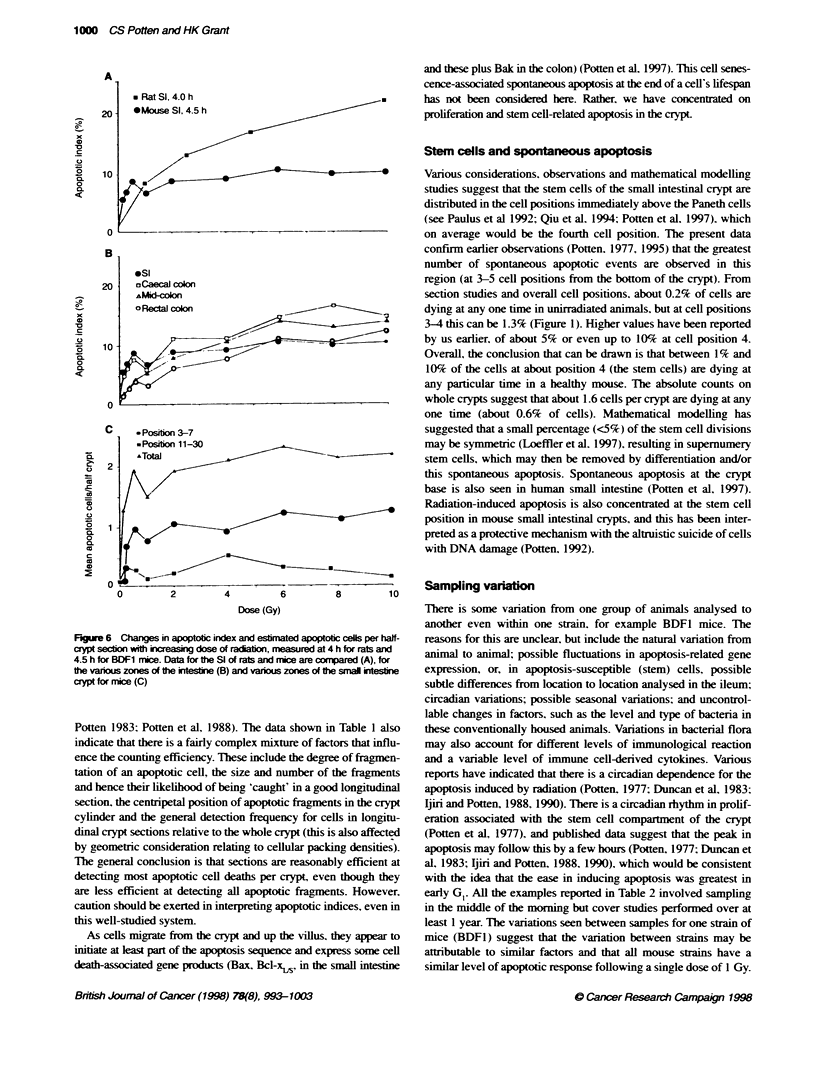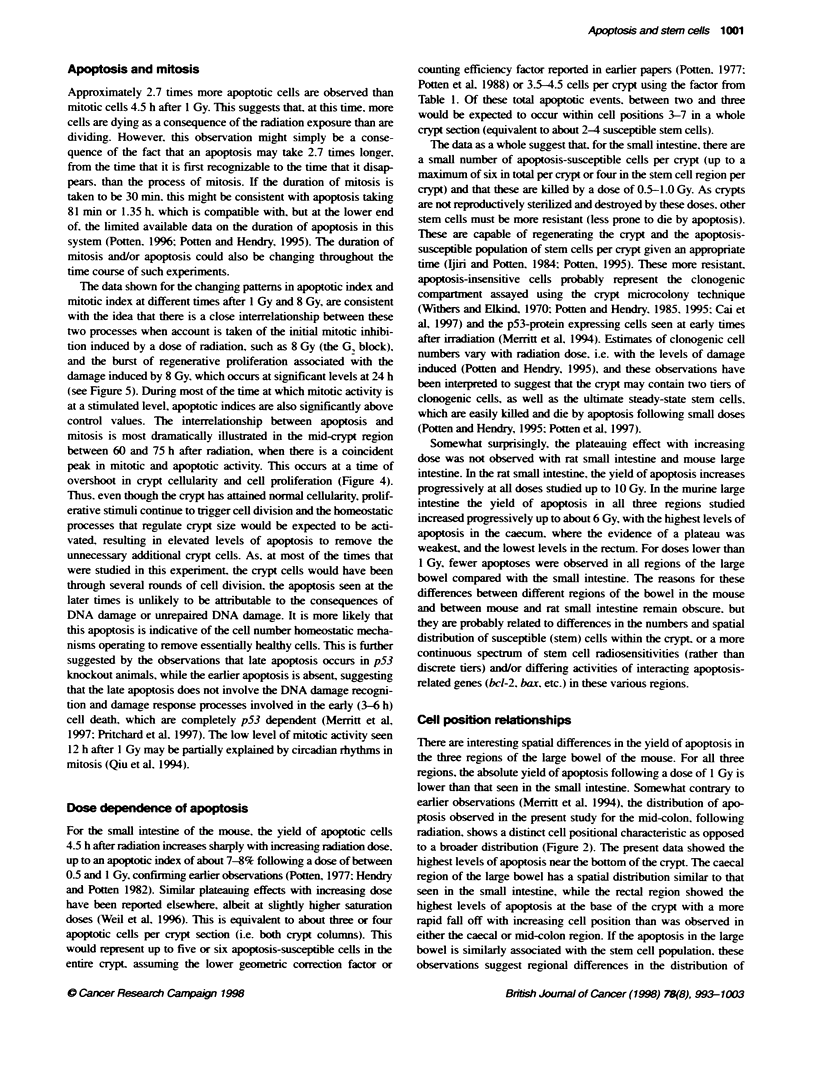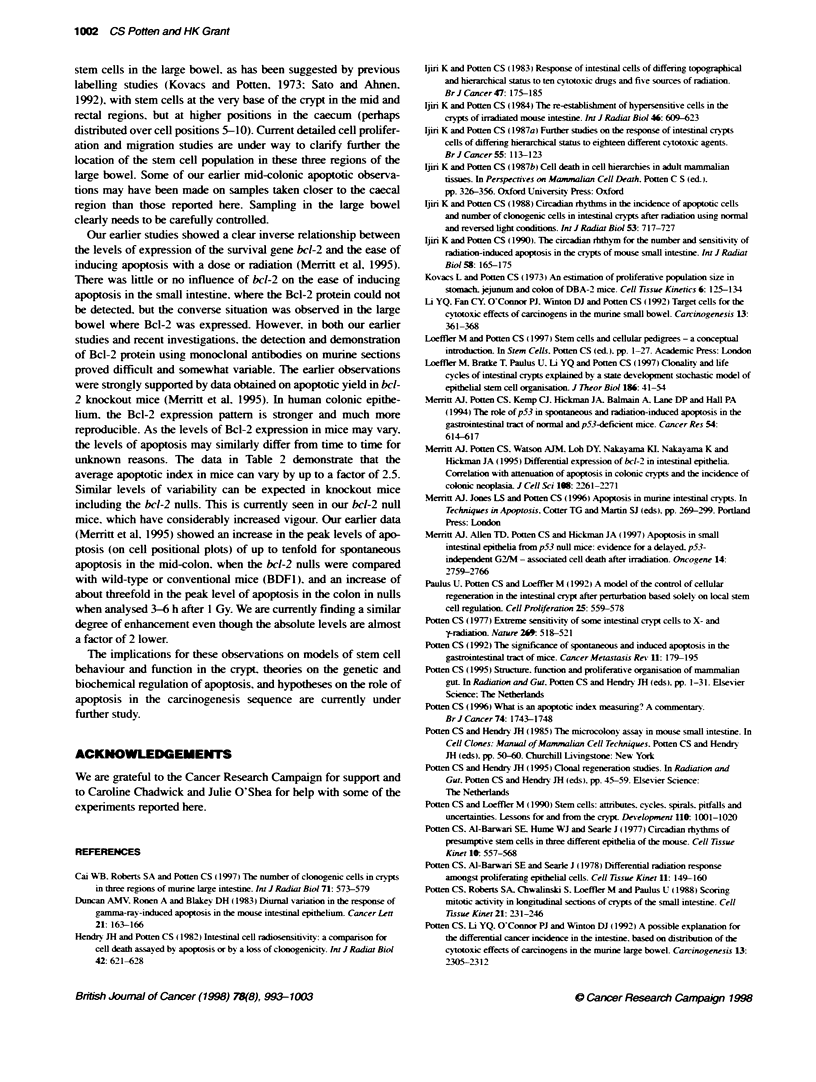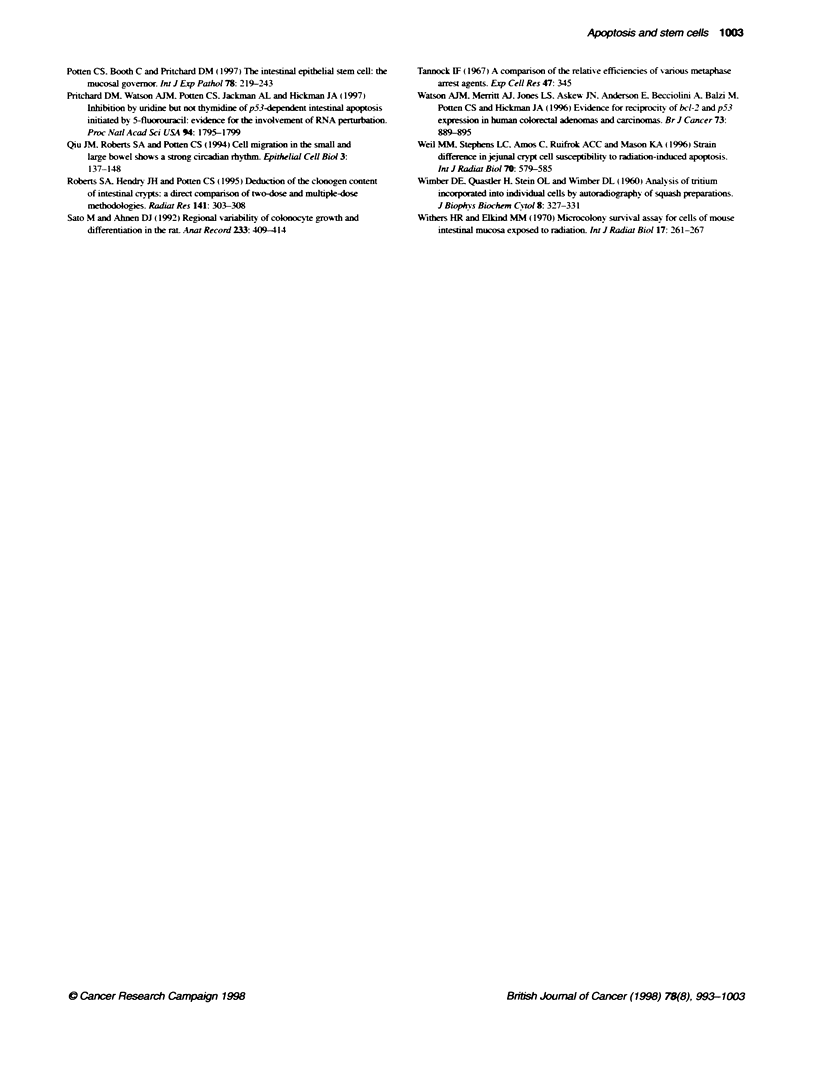# The relationship between ionizing radiation-induced apoptosis and stem cells in the small and large intestine.

**DOI:** 10.1038/bjc.1998.618

**Published:** 1998-10

**Authors:** C. S. Potten, H. K. Grant

**Affiliations:** Epithelial Biology CRC Group, Paterson Institute for Cancer Research, Christie Hospital NHS Trust, Manchester, UK.

## Abstract

Apoptosis is observed in the crypts of the small intestine of healthy animals and man (spontaneous apoptosis). The levels can be dramatically elevated 3-6 h following ionizing radiation exposure. Both the spontaneous and radiation-induced apoptosis in the small intestine crypts are most frequently observed at the positions in the crypt associated with stem cells (about four cell positions from the base of the crypt). The number of apoptotic deaths can be counted in routine histological preparations, but interpretation of the counts is complicated by numerous factors. However, recording the number of cells containing one or more apoptotic fragments in crypt sections provides a good estimate for the absolute number of cell deaths in crypts. Similarities are noted in the frequency and cell positional relationship of radiation-induced apoptosis in the small intestine of various strains of mice and one strain of rat. Apoptosis in the large intestine is generally lower in frequency than in the small intestine and, for the mid-colonic and rectal regions, has a different cell positional frequency distribution, with the highest apoptotic yield at the crypt base. The caecal colon has a pattern of apoptotic distribution more similar to that in the small intestine. After exposure to 1 Gy ionizing radiation, the maximum apoptotic yield occurs over a period of 3-6 h in the small intestine. There is some unexplained variability in the values between groups of mice and between different mouse strains. After 8 Gy, the yield remains elevated for several days, however a similar maximum yield is still observed at the early times. In mouse large intestine and rat small intestine, the yield continues to rise until about 6 Gy in mouse large intestine and until at least 10 Gy in rat small intestine. Spontaneous apoptosis is interpreted as part of the homeostatic mechanism regulating stem cell numbers. About 1.6 cells per crypt are dying at any one time. Following irradiation, there is an apparent relationship between mitotic and apoptotic levels, suggesting that these processes are linked. The dose-response relationship suggests that there are about six apoptosis-susceptible cells in crypts of the small intestine, with about 2-4 of these occurring at cell positions in which there are other more resistant clonogenic cells. In the large intestine, the position of these apoptosis-susceptible cells varies with region, but the numbers are similar.


British Journal of Cancer (1998) 78(8), 993–1003
© 1998 Cancer Research Campaign

### The relationship between ionizing radiation-induced apoptosis and stem cells in the small and large intestine

**CS Potten and HK Grant**

Epithelial Biology CRC group, Paterson Institute for Cancer Research, Christie Hospital NHS Trust, Wilmslow Road, Manchester M20 4BX, UK

**Summary** Apoptosis is observed in the crypts of the small intestine of healthy animals and man (spontaneous apoptosis). The levels can be dramatically elevated 3–6 h following ionizing radiation exposure. Both the spontaneous and radiation-induced apoptosis in the small intestine crypts are most frequently observed at the positions in the crypt associated with stem cells (about four cell positions from the base of the crypt). The number of apoptotic deaths can be counted in routine histological preparations, but interpretation of the counts is complicated by numerous factors. However, recording the number of cells containing one or more apoptotic fragments in crypt sections provides a good estimate for the absolute number of cell deaths in crypts. Similarities are noted in the frequency and cell positional relationship of radiation-induced apoptosis in the small intestine of various strains of mice and one strain of rat. Apoptosis in the large intestine is generally lower in frequency than in the small intestine and, for the mid-colonic and rectal regions, has a different cell positional frequency distribution, with the highest apoptotic yield at the crypt base. The caecal colon has a pattern of apoptotic distribution more similar to that in the small intestine. After exposure to 1 Gy ionizing radiation, the maximum apoptotic yield occurs over a period of 3–6 h in the small intestine. There is some unexplained variability in the values between groups of mice and between different mouse strains. After 8 Gy, the yield remains elevated for several days, however a similar maximum yield is still observed at the early times. In mouse large intestine and rat small intestine, the yield continues to rise until about 6 Gy in mouse large intestine and until at least 10 Gy in rat small intestine. Spontaneous apoptosis is interpreted as part of the homeostatic mechanism regulating stem cell numbers. About 1.6 cells per crypt are dying at any one time. Following irradiation, there is an apparent relationship between mitotic and apoptotic levels, suggesting that these processes are linked. The dose–response relationship suggests that there are about six apoptosis-susceptible cells in crypts of the small intestine, with about 2–4 of these occurring at cell positions in which there are other more resistant clonogenic cells. In the large intestine, the position of these apoptosis-susceptible cells varies with region, but the numbers are similar.

**Keywords:** ionizing radiation; apoptosis; intestinal crypts; stem cells; small intestine; large intestine

Apoptosis occurs in the crypts of the small intestine of healthy mice (Potten, 1977, 1995) at a higher level than in the colon. In the small intestine, this spontaneous apoptosis, observed in routine paraffin sections cut longitudinally through the crypts, occurs predominantly at the position in the crypts associated with stem cells, i.e. about the fourth cell position from the base of the crypt immediately above the Paneth cells (Potten and Loeffler, 1990; Loeffler and Potten, 1997; Potten et al, 1997). This spontaneous apoptosis is about tenfold less common in the large bowel and is not particularly associated with the positions linked with stem cells (cell positions 1–2 at the base of the crypt). This absence of cell death in the stem cell location in crypts in the large bowel is believed to be associated with the active expression of *bcl-2*, seen at these positions in both mouse and man (Merritt et al, 1995).

Apoptosis can easily be recognized and quantified in this well-studied system when appropriately stained sections are cut from rapidly fixed tissue. The levels of apoptosis in the crypts can be raised by exposure to a variety of cytotoxic agents (Ijiri and Potten, 1985, 1987*a,b*; Li et al, 1992; Potten et al, 1992). Ionizing radiation and certain cytotoxic drugs and chemical mutagens induce apoptosis specifically in the stem cell positions of the small bowel, with the maximum yields of apoptosis being observed within a period of 3–6 h after exposure.

Following radiation, levels of wild-type *p53* expression are elevated, with a time course in the small intestine similar to that for apoptosis, i.e. peak levels are observed 3–6 h after irradiation (Merritt et al, 1994). The number of cells strongly reactive to an antibody to wild-type *p53* (CM5) and the position of these positive-staining nuclei in the crypt very closely resemble the yield and distribution of apoptosis (Merritt et al, 1994). However, apoptotic cells are rarely positive for p53 protein, at least for the time points studied and immunohistochemical approaches used. The p53-positive cells, which occur at a similar location in the crypt to the apoptotic cells, must represent other stem cells that survive the cytotoxic exposure, possibly those subject to a cell cycle checkpoint regulated by p53 and $p21^{waf-1/cip1}$. The resultant cell cycle arrest would allow time for repair of DNA damage, resulting in a greater resistance to radiation damage. They are, therefore, likely to be a part of the more resistant clonogenic stem cell compartment (Potten and Hendry, 1995; Roberts et al, 1995; Potten et al, 1997).

Such studies have resulted in the formulation of an hypothesis that spontaneous apoptosis is an important process involved in the homeostatic regulation of stem cell numbers in the undamaged small intestinal crypt and that, following DNA damage, apoptosis

*Received 3 November 1997*
*Revised 11 February 1998*
*Accepted 17 February 1998*

*Correspondence to:* CS Potten is an important protective mechanism removing the cells bearing the damage and, hence, the damage itself (Potten, 1992; Potten et al, 1992). In the large intestine, both the spontaneous apoptosis and radiation-induced apoptosis are suppressed, probably because of the action of *bcl*-2, which is, however, only one of a large family of interacting 'survival' and 'death' genes. As a consequence of *bcl*-2, the homeostatic mechanisms governing stem cell numbers may be more relaxed in the colon and stem cell numbers may tend to increase with the passage of time. This would result in more carcinogen target cells at risk and occasional hyperplastic crypts. The DNA damage-induced apoptosis is also suppressed in the colon. Thus, the protective apoptosis that operated in the small intestine to remove damaged cells is less efficiently operated thereby increasing the risk that DNA damage may be perpetuated in the large intestinal stem cells. This hypothesis provides a new explanation for the well-known differential human cancer incidence figures for the small and large intestine. The hypothesis was further supported by observations in *bcl*-2 knockout mice, in which the levels of both spontaneous and radiation-induced apoptosis in the large intestine were greatly elevated in the stem cell location. In contrast, the removal of the *bcl*-2 gene had no effect on the levels of spontaneous or radiation-induced apoptosis in the small bowel, in which *bcl*-2 expression was absent (Merritt et al, 1995).

These studies, and the hypothesis derived from the observations, are dependent on labour intensive quantitative analysis of histological sections of the intestine. As a consequence, the extent to which these observations apply to other strains of mice and other species and different regions of the small and large bowel remain unknown, although observations on the limited material that can be obtained from human subjects do support the hypothesis (Merritt et al, 1995; Watson et al, 1996). Here, we present more extensive data on different strains of mice and one strain of rat. We have also analysed the pattern of radiation-induced apoptosis in different regions of the BDF1 murine large intestine. This was performed specifically to address the possibility that the apoptosis reaction may be related to, or some expression of, the stem cell population; and that the spatial distribution of apoptosis and also stem cells may differ in the different regions of the large bowel, as has been suggested by some DNA labelling experiments (Kovacs and Potten, 1973; Sato and Ahnen, 1992).

### MATERIALS AND METHODS

### Animals

The bulk of our previous studies were performed in male BDF1 mice aged between 10 and 12 weeks, bred and maintained within conventional housing conditions at the Paterson Institute with food and water ad libitum and a 12-h light cycle (lights on at 06.00 h). These represent our standard experimental laboratory mice, and many of the experiments reported here have involved the use of these animals. In addition, comparably aged male mice of the following strains have been studied: C3H/He, C57, DBA2, 129, Balb-c, CBA, AKR and homozygous wild-type animals for various transgenic knockout strains. Some studies have also been performed on outbred Wistar rats with a body weight of approximately 200 g. A minimum of four mice or rats have always been used as an experimental group. Some experimental groups have been repeated up to six times within the course of the present experiments, providing pooled groups of up to 24–30 animals. All experiments have been performed within the regulations of the United Kingdom Animals (Scientific Procedures) Act 1986.

### Radiation

Groups of mice were whole body irradiated with either 1 Gy or 8 Gy of caesium gamma-rays delivered at a dose rate of 3.8 Gy $min^{-1}$. The mice were not anaesthetized during irradiation and received air pumped into the radiation chamber. Rats received a dose of 1 Gy of 300 kVp X-rays (HVL 2.3 mm of copper) delivered to the entire body.

In one experiment, animals received a range of doses up to 10 Gy, with groups of animals being killed 4.5 h after radiation ($\gamma$-rays for mice, X-rays for rats).

### Fixation

Groups of animals were killed by anaesthetic overdose or cervical dislocation usually 4.5 h after radiation (which for BDF1 mice represents the time of maximum yield of apoptotic fragments in the small intestinal crypt) (Potten, 1977; Potten et al, 1978; Hendry and Potten, 1982). However, in one experiment, groups of animals were killed at varying times after exposure to radiation. The small intestine and large intestine were rapidly removed from the abdomen and the intestinal contents were gently eased out of the intestinal tube prior to fixation in Carnoy's fixative for 20–30 min. During this time, the intestines remained intact and were fixed flat on filter paper in a Petri dish. After the initial fixation, the tissue was transferred to 70% ethanol for storage before processing for histology. The intestine was cut into approximately ten 1-cm lengths which were bundled together in micropore tape, embedded and sectioned at 3–5 µm as described previously (Potten and Hendry, 1985).

The large bowel was divided into three approximately equal lengths, referred to for convenience as caecum, mid-colon and rectum. In each region, segments were bundled as described above. The segment of small intestine analysed was predominantly terminal ileum. Up to ten intestinal cross-sections were obtained from each bundle (each animal), among which it was not possible to ascribe a specific position along the region of gut.

In a few groups, other segments of ileum were fixed in Clarke's fixative, hydrolysed in 5 N hydrochloric acid and stained with Schiff's reagent. From these samples, intact crypts were dissociated and mounted on microscope slides. This represents a slight modification of the crypt squash technique (Wimber et al, 1960; Potten et al, 1988).

### Microscopical analysis

The haematoxylin and eosin (H + E)-stained sections and whole crypt preparations were analysed on a Zeiss microscope using a ×40 planapo oil objective. Crypts were selected for scoring if they represented good longitudinal sections containing crypt lumen, Paneth cells and more than 17 cells in the crypt column. For the whole isolated crypts, crypts were selected which could be optically focused at all levels, so that all cells could be detected. One side (one crypt column) of 50 longitudinal crypt sections per mouse or 50 whole crypts per mouse were selected for counting.

For the crypt sections, the presence or absence of one or more apoptotic fragments at each sequential cell position in the crypt column, starting at the midpoint at the base of the crypt, was recorded using our own software. The number of mitotic cells was recorded in a similar cell positional fashion at the same time. For the whole crypt preparations, the crypt was carefully sectioned optically through its entirety, recording the number of mitotic cells

© Cancer Research Campaign 1998

**Table 1** Characterization of apoptotic counting in BDF1 mice

| | Sections one side of crypt section | | | Whole crypts | | | | 200 half-crypt sections to whole crypt comparisons | | |
|---|---|---|---|---|---|---|---|---|---|---|
| | Total[a,e] fragments | Apoptotic cells[f] | AI [%[b,f] (cells)] | Total fragments | Apoptotic cells[d] | AI [%[c,d] (cells)] | Fragments/ apoptosis | Apoptotic cells* | Apoptotic fragments* | AI (%) |
| Control irradiated | 68 | 64 | 1.1 | 164 | 78 | 0.2 | 2.1 | 0.82 | 0.41 | 5.5 |
| 1 Gy 3 h | 551 | 398 | 7.1 | 4024 | 1233 | 2.5 | 3.3 | 0.32 | 0.14 | 2.8 |
| 9 h | 549 | 371 | 6.6 | 2414 | 807 | 1.6 | 3.0 | 0.46 | 0.23 | 4.1 |
| 8 Gy 3 h | 370 | 260 | 4.6 | 3302 | 1225 | 2.5 | 2.7 | 0.21 | 0.11 | 1.8 |
| 9 h | 562 | 392 | 7.0 | 2592 | 1098 | 2.2 | 2.4 | 0.36 | 0.22 | 3.2 |
| Mean | | | | | | | 2.7 | 0.43* | 0.22* | 3.5 |

[a]Number in one crypt column, i.e. up one side of a crypt. [b]In the small intestine about 48% of all fragments in controls are found over cell positions 1–6 (see Figure 1). About 59% of all fragments 4.5 h after 1 Gy are found over cell positions 1–6. [c]On average, there are about 250 cells per crypt [(apoptotic cells ÷ 200 × 250) × 100]. [d]An apoptotic cell corresponds to a cluster of apoptotic fragments. [e]In the sections the number of fragments adjacent to each cell position was recorded, therefore giving a total number of fragments. [f]This number corresponds to the number of cell positions with one or more adjacent apoptotic fragments. On average there are about 28 cells per crypt column, i.e. about 28 cell positions [(apoptotic cells ÷ 200 × 28) × 100]. *0.86 and 0.44 when both sides of the crypt section are considered. All numbers in this column should be similarly doubled for a whole crypt section.

**Figure 1** Data are presented for the levels of fragmentation during apoptosis, the number of apoptotic events per crypt and the apoptotic index for the small intestine (SI) of BDF1 male mice, unirradiated or 4.5 h after 1 Gy of γ-rays. A minimum of 50 crypt sections (apoptotic index) or 50 whole crypts (fragments/apoptosis or apoptoses per crypt) were scored per mouse with a minimum of four mice per group. The number of mice is shown in brackets when it is more than four. The right-hand graphs show typical cell position frequency plots as bar diagrams or as a smoothed curve.

Table 2 Apoptotic and mitotic indices in small and large intestine in various mouse strains[a] (1 Gy 4.5 h) and rats

| Strain | Small intestine | | | Large intestine | | |
|---|---|---|---|---|---|---|
| | AI (%) | MI (%) | AI/MI | AI (%) | MI (%) | AI/MI |
| BDF1 | 6.6 | 2.5 | 2.6 | 5.2 | 2.0 | 2.6 |
| | 7.8 | 3.2 | 2.4 | 4.8 | 1.2 | 4.0 |
| | 10.0 | 3.6 | 2.8 | 6.3 | 1.8 | 3.5 |
| | 8.9 | 2.5 | 3.6 | | | |
| | 4.0 | 2.6 | 1.5 | | | |
| | 6.6 | 1.8 | 3.7 | | | |
| | 9.4 | 3.4 | 2.8 | | | |
| Mean ± s.d. | 7.6 ± 2.1 | 2.8 ± 0.6 | 2.8 ± 0.7 | 5.4 ± 0.7 | 1.7 ± 0.4 | 3.4 ± 0.7 |
| *p53* +/+[b] | 4.3 | 2.6 | 1.7 | 2.6 | 1.2 | 2.2 |
| nu/nu[c] | 7.0 | 3.1 | 2.2 | | | |
| DBA-2 | 6.7 | 3.1 | 2.2 | 4.1 | 1.2 | 3.4 |
| CBA | 5.2 | 2.0 | 2.6 | 4.0 | 0.8 | 5.0 |
| AKR | 7.6 | 1.9 | 4.0 | 5.6 | 1.1 | 5.1 |
| 129 | 6.0 | 1.5 | 4.0 | 1.7 | 0.9 | 1.9 |
| C3H/He | 6.9 | 1.5 | 4.6 | 3.4 | 0.5 | 6.8 |
| Balb/c | 8.6 | 1.6 | 5.4 | 3.3 | 0.7 | 4.7 |
| C57 | 11.6 | 2.1 | 5.5 | 4.1 | 1.3 | 3.2 |
| Mean ± s.d. | 7.1 ± 2.1 | 2.2 ± 0.6 | 3.6 ± 1.4 | 3.6 ± 1.2 | 1.0 ± 0.3 | 4.0 ± 1.7 |
| Wistar rats | 8.2 | 3.1 | 2.6 | 3.1 | 2.0 | 1.6 |

[a]Ranked according to AI/MI for small intestine. Ileum and mid colon analysed. Four mice. 200 half-crypt sections analysed per group. [b]On a 129 background. [c]On a Balb-c background.

and the number of apoptotic cells. The latter were judged subjectively by the size and number of closely adjacent apoptotic fragments. The number of fragments was also counted. An apoptotic cell could be judged as a single large fragment approximately the size of a neighbouring cell or a cluster of closely associated small fragments together constituting an area comparable to a neighbouring cell. Comparative studies have been performed using in situ end labelling (ISEL) or TUNEL techniques which, in this tissue, show both false positives and false negatives when compared with morphology-based assessment of apoptosis. As a consequence, we prefer to assess apoptosis morphologically (Merritt et al, 1996).

### Data presentation

The data are presented in tabular form or as cell position frequency plots. These have been smoothed using a running average of three positions to ease analysis and comparisons.

### RESULTS

The counting efficiency of sectioned material can be assessed by comparing section data with data from whole crypts which represent the absolute counts (see Table 1).

The recording of whether or not an apoptotic body occurs at each position along the crypt column slightly overestimates (by 1.64-fold) the true number of cell deaths that occur in untreated control animals and slightly underestimates (by 0.64-fold) the true number in an irradiated animal (see column 9 in Table 1) (the overall average is 0.86-fold). The reason for this difference is unclear, but may be due to differences in the degree of fragmentation, size and removal processes for spontaneous and radiation-induced apoptosis. Overall, the quantitation of apoptosis by counting the number of cells that contain one or more apoptotic fragments provides a reasonable estimate of the number of cells dying by apoptosis in a crypt.

Sections are less efficient at detecting all the apoptotic fragments (only 44% of the total fragments are detected). This is probably because the fragments are considerably smaller than an average cell nucleus in size and an underestimation in their numbers results, because there will be a smaller likelihood of sectioning small fragments (a Tannock type of size correction in reverse would be required, Tannock, 1967; Potten et al, 1988). The major problem is seen in the difference between the apoptotic index (AI) in sections and in whole crypts. Sections provide values that are on average 3.5-fold higher for the AI. Sections detect 86% of all apoptotic cells but record only about 10% of all the crypt cells (8.9% using the figures in Table 1). These values, when taken in conjunction with earlier estimates that take into account size and centripetal position of apoptotic events relative to the cell nuclei used to count cells, probably explain the discrepancy. The AI determined from whole crypts should be taken as the most reliable value.

In the whole crypt, the absolute number of apoptotic cell deaths can be determined, as can the number of fragments generated by each dying apoptotic cell. The average number of fragments per apoptosis is 2.7 (range 2.1–3.3, as shown in Table 1 and illustrated in Figure 1). The maximum number of fragments per apoptosis can be up to 12 following radiation.

As can be seen from the data obtained for whole crypts (Table 1), about 0.2% of the crypt cells are dying in normal healthy unirradiated BDF1 mice. This percentage is raised eight- to 12.5-fold (about ten fold) at early times following irradiation with either 1 Gy or 8 Gy; 8 Gy does not result in significantly more apoptotic cells at these early times than 1 Gy.

At 3 h following 1 Gy, 6.2 apoptotic cell deaths can be expected in each crypt (Figure 1) (or 5.5 at 3 h in Table 1). However, the range is again broad from zero or one apoptotic event per crypt through to 12. Thus, it is very important to count sufficient crypts. Both the spontaneous and radiation-induced apoptosis is seen most frequently at around the third to sixth positions in the crypt (see Figure 1), where the AI may be 1.3% in controls for cell position 3

 © Cancer Research Campaign 1998

**Figure 2** Cell positional frequency plots (smoothed over three cell positions) for BDF1 mice and Wistar rats for small intestine (SI) and mid colon (MC) 4.5 or 4.0 h after 1 Gy. Data for three regions of the large intestine of BDF1 are also presented plus 4 (22% of all apoptoses are found at these two cell positions) and 16.7% at 4.5 h after 1 Gy (18% of all apoptoses are found at these two cell positions and 40% over cell positions 3–6). At 4.5 h after 1 Gy in sections, the apoptotic index is on average 7.6% and the mitotic index 2.8%, i.e. 2.7 times more apoptotic figures are seen than mitotic figures (Table 2).

As can be seen in Table 2, the average values obtained from groups of four male BDF1 mice, analysed at various times over a

**Figure 3** Changes in the apoptotic index for the crypt as a whole (A and C), lower ten cell positions (B) and cell positions 11–16 (D) at 4.5 h after 1 Gy. Data are presented for various regions of intestine from BDF1 mice and Wistar rats

2-year period, can range between 4.0 and 10.0 for the apoptotic index (mean 7.6) and between 1.8 and 3.6 for the mitotic index at 4.5 h after 1 Gy. The cell positional patterns are shown in Figure 2 together with data for rats. The values in the large bowel for both apoptotic index and mitotic index are consistently between 60% and 70% of those in the small intestine.

A similar range of apoptotic index values have been observed for a variety of mouse strains exposed to 1 Gy analysed at the same time (Table 2). The range in apoptotic indices here was 4.3–11.6 with an overall mean value of 7.1, which is very similar to the variation and overall mean value for BDF1 mice. However, in some of the strains there was a tendency for the mitotic figures to be lower and, as a consequence, the ratio of apoptotic index to mitotic index (AI/MI) tended to be higher (rising from 1.7 to 5.5). Again in these other strains, the apoptotic and mitotic indices in the large bowel were lower than in the small intestine by 50% and 45% respectively. The cell positional distributions for the strains generally resembled those shown in Figure 2, with the maximum yield of apoptosis being observed in the stem cell zone of the crypt of the small intestine at around cell positions 3–5. The yield of apoptosis in the colon of the 129 strain and in the *p53* wild-type animals (+/+), which were originally derived from 129, is lower than seen in all other strains.

Following a dose of 1 Gy, the maximum yield of apoptosis observed in the small intestine and the large intestine generally occurs somewhat variably between 3 and 6 h. At longer time intervals, the apoptotic index declines. The data for small intestine and the mid-colon region of the large intestine are shown in Figure 3, combined with observations for other regions for the large bowel (caecal and rectal zones) and for the small and large intestine of the rat.

Following a dose of 8 Gy to BDF1 mice, similar values were observed at the time of the peak, i.e. 3–6 h, as was shown in Tables 1 and 2 for 1 Gy. However, the decline in apoptotic cells with time is delayed after 8 Gy, and high levels of apoptosis can still be observed at 24 h. At the early times mitosis is completely suppressed, while at later times it is elevated relative to control values because of the compensatory regenerative response, which is particularly pronounced at around 60–100 h, when it overshoots the control value (Figure 4). The changing relationship between apoptosis and mitosis is dramatically illustrated by the ratio AI/MI shown in Figure 5. At 24 h after 8 Gy, approximately 12 times more apoptotic cells are observed than mitotic cells. Between 60 and 72 h, the number of apoptotic and mitotic cells are approximately equal (AI/MI = 1.0), while between 75 and 120 h, about 1.28 times more mitotic figures are seen than apoptotic cells.

The changing values of the apoptotic index and mitotic index for the times beyond 24 h are shown in Figure 4 for the crypt as a whole and for the cells in the lower half and the upper half of the crypt separately. These data suggest a relationship between mitotic activity and apoptotic activity. This is evident in the mid-crypt region (cp 11–16), where a burst of mitotic activity is seen to occur at around 60–75 h and this is accompanied by a small burst in apoptotic activity. Figure 5 shows representative examples of cell positional plots at various times after 8 Gy. At 4.5 h apoptosis is near maximum, while mitosis is abolished (due to the $G_2$ block).

© Cancer Research Campaign 1998

**Figure 4** Changes in apoptotic index (left scale and solid line) and mitotic index (right scale and dashed line) with time after 8 Gy. Control values are shown by the respective horizontal lines. (A) Values for the crypt as a whole. (B) data for cell positions 1–10 and (C) data for cell positions 11–16

**Figure 5** The changing pattern in the ratio of apoptosis to mitosis (AI/MI) at various times after 1 Gy (closed circles) and after 8 Gy (closed squares) for BDF1 small intestine (A). The other two graphs show the cell positional changes in apoptotic (B) and mitotic (C) index with time after 8 Gy. Peaks in mitotic activity are seen at cell positions 5 at 48 h. cell position 9 at 60 h and cell position 11 at 65 h

At 24 h. regenerative proliferation (mitosis) is beginning at the crypt base (peak at Cp5). There is a late wave of apoptosis at this time. At 48 h there is still a stem cell-associated mitotic peak. but apoptosis is declining and appears higher up the crypt. At later times. apoptosis declines further. while mitosis remains high. with the peak mitotic activity shifting from about cell position 5 at 48 h to cell position 9 at 60 h. 11 at 65 h and showing a very broad peak over many cell positions at 80 h.

Analyses of the changing yield of apoptotic cells observed in BDF1 crypt sections at 4.5 h after exposure to different doses of radiation are shown in Figure 6. The yield of apoptotic cells tends to plateau in the small intestine at between 0.5 and 1 Gy. As has been suggested before (Potten. 1977: Hendry and Potten. 1982). it is difficult to distinguish between a true plateau and a decreased slope in the dose–response at doses beyond 1 Gy. As can be seen from the data shown in Figure 6. in the rat small intestine this plateauing effect is not apparent. In the mouse. the yield of apoptotic cells observed in the mid-colon and rectum is lower at a given dose than in the small intestine when doses below about 2–4 Gy are considered. However. the incidence of cell death continues to rise in all three regions of the mouse large intestine up to doses of about 6 Gy. Thereafter. a plateau. or a more shallow increase in apoptotic yield with dose. can be observed. At all doses in the small bowel of the mouse. the greatest proportion of cell death is observed in the lower half of the crypt – as can be seen in Figure 6.

### DISCUSSION

### Apoptotic scoring

The data presented here demonstrate that the detection efficiency of cell death via apoptosis using section material is high. and this has been validated by comparing the absolute number of apoptotic cells in whole crypts. optically sectioned at all levels. with counts of cells containing one or more apoptotic fragments in sections. The detection efficiency reported here is similar to those quoted in earlier papers using less detailed analyses (Potten. 1977: Ijiri and

© Cancer Research Campaign 1998 

**Figure 6** Changes in apoptotic index and estimated apoptotic cells per half-crypt section with increasing dose of radiation, measured at 4 h for rats and 4.5 h for BDF1 mice. Data for the SI of rats and mice are compared (A), for the various zones of the intestine (B) and various zones of the small intestine crypt for mice (C)

Potten 1983; Potten et al, 1988). The data shown in Table 1 also indicate that there is a fairly complex mixture of factors that influence the counting efficiency. These include the degree of fragmentation of an apoptotic cell, the size and number of the fragments and hence their likelihood of being 'caught' in a good longitudinal section, the centripetal position of apoptotic fragments in the crypt cylinder and the general detection frequency for cells in longitudinal crypt sections relative to the whole crypt (this is also affected by geometric consideration relating to cellular packing densities). The general conclusion is that sections are reasonably efficient at detecting most apoptotic cell deaths per crypt, even though they are less efficient at detecting all apoptotic fragments. However, caution should be exerted in interpreting apoptotic indices, even in this well-studied system.

As cells migrate from the crypt and up the villus, they appear to initiate at least part of the apoptosis sequence and express some cell death-associated gene products (Bax, Bcl-$x_{L/S}$, in the small intestine and these plus Bak in the colon) (Potten et al, 1997). This cell senescence-associated spontaneous apoptosis at the end of a cell's lifespan has not been considered here. Rather, we have concentrated on proliferation and stem cell-related apoptosis in the crypt.

### Stem cells and spontaneous apoptosis

Various considerations, observations and mathematical modelling studies suggest that the stem cells of the small intestinal crypt are distributed in the cell positions immediately above the Paneth cells (see Paulus et al 1992; Qiu et al, 1994; Potten et al, 1997), which on average would be the fourth cell position. The present data confirm earlier observations (Potten, 1977, 1995) that the greatest number of spontaneous apoptotic events are observed in this region (at 3–5 cell positions from the bottom of the crypt). From section studies and overall cell positions, about 0.2% of cells are dying at any one time in unirradiated animals, but at cell positions 3–4 this can be 1.3% (Figure 1). Higher values have been reported by us earlier, of about 5% or even up to 10% at cell position 4. Overall, the conclusion that can be drawn is that between 1% and 10% of the cells at about position 4 (the stem cells) are dying at any particular time in a healthy mouse. The absolute counts on whole crypts suggest that about 1.6 cells per crypt are dying at any one time (about 0.6% of cells). Mathematical modelling has suggested that a small percentage (<5%) of the stem cell divisions may be symmetric (Loeffler et al, 1997), resulting in supernumery stem cells, which may then be removed by differentiation and/or this spontaneous apoptosis. Spontaneous apoptosis at the crypt base is also seen in human small intestine (Potten et al, 1997). Radiation-induced apoptosis is also concentrated at the stem cell position in mouse small intestinal crypts, and this has been interpreted as a protective mechanism with the altruistic suicide of cells with DNA damage (Potten, 1992).

### Sampling variation

There is some variation from one group of animals analysed to another even within one strain, for example BDF1 mice. The reasons for this are unclear, but include the natural variation from animal to animal; possible fluctuations in apoptosis-related gene expression, or, in apoptosis-susceptible (stem) cells, possible subtle differences from location to location analysed in the ileum; circadian variations; possible seasonal variations; and uncontrollable changes in factors, such as the level and type of bacteria in these conventionally housed animals. Variations in bacterial flora may also account for different levels of immunological reaction and a variable level of immune cell-derived cytokines. Various reports have indicated that there is a circadian dependence for the apoptosis induced by radiation (Potten, 1977; Duncan et al, 1983; Ijiri and Potten, 1988, 1990). There is a circadian rhythm in proliferation associated with the stem cell compartment of the crypt (Potten et al, 1977), and published data suggest that the peak in apoptosis may follow this by a few hours (Potten, 1977; Duncan et al, 1983; Ijiri and Potten, 1988, 1990), which would be consistent with the idea that the ease in inducing apoptosis was greatest in early $G_1$. All the examples reported in Table 2 involved sampling in the middle of the morning but cover studies performed over at least 1 year. The variations seen between samples for one strain of mice (BDF1) suggest that the variation between strains may be attributable to similar factors and that all mouse strains have a similar level of apoptotic response following a single dose of 1 Gy.

© Cancer Research Campaign 1998

### Apoptosis and mitosis

Approximately 2.7 times more apoptotic cells are observed than mitotic cells 4.5 h after 1 Gy. This suggests that, at this time, more cells are dying as a consequence of the radiation exposure than are dividing. However, this observation might simply be a consequence of the fact that an apoptosis may take 2.7 times longer, from the time that it is first recognizable to the time that it disappears, than the process of mitosis. If the duration of mitosis is taken to be 30 min, this might be consistent with apoptosis taking 81 min or 1.35 h, which is compatible with, but at the lower end of, the limited available data on the duration of apoptosis in this system (Potten, 1996; Potten and Hendry, 1995). The duration of mitosis and/or apoptosis could also be changing throughout the time course of such experiments.

The data shown for the changing patterns in apoptotic index and mitotic index at different times after 1 Gy and 8 Gy, are consistent with the idea that there is a close interrelationship between these two processes when account is taken of the initial mitotic inhibition induced by a dose of radiation, such as 8 Gy (the $G_2$ block), and the burst of regenerative proliferation associated with the damage induced by 8 Gy, which occurs at significant levels at 24 h (see Figure 5). During most of the time at which mitotic activity is at a stimulated level, apoptotic indices are also significantly above control values. The interrelationship between apoptosis and mitosis is most dramatically illustrated in the mid-crypt region between 60 and 75 h after radiation, when there is a coincident peak in mitotic and apoptotic activity. This occurs at a time of overshoot in crypt cellularity and cell proliferation (Figure 4). Thus, even though the crypt has attained normal cellularity, proliferative stimuli continue to trigger cell division and the homeostatic processes that regulate crypt size would be expected to be activated, resulting in elevated levels of apoptosis to remove the unnecessary additional crypt cells. As, at most of the times that were studied in this experiment, the crypt cells would have been through several rounds of cell division, the apoptosis seen at the later times is unlikely to be attributable to the consequences of DNA damage or unrepaired DNA damage. It is more likely that this apoptosis is indicative of the cell number homeostatic mechanisms operating to remove essentially healthy cells. This is further suggested by the observations that late apoptosis occurs in *p53* knockout animals, while the earlier apoptosis is absent, suggesting that the late apoptosis does not involve the DNA damage recognition and damage response processes involved in the early (3–6 h) cell death, which are completely *p53* dependent (Merritt et al, 1997; Pritchard et al, 1997). The low level of mitotic activity seen 12 h after 1 Gy may be partially explained by circadian rhythms in mitosis (Qiu et al, 1994).

### Dose dependence of apoptosis

For the small intestine of the mouse, the yield of apoptotic cells 4.5 h after radiation increases sharply with increasing radiation dose, up to an apoptotic index of about 7–8% following a dose of between 0.5 and 1 Gy, confirming earlier observations (Potten, 1977; Hendry and Potten 1982). Similar plateauing effects with increasing dose have been reported elsewhere, albeit at slightly higher saturation doses (Weil et al, 1996). This is equivalent to about three or four apoptotic cells per crypt section (i.e. both crypt columns). This would represent up to five or six apoptosis-susceptible cells in the entire crypt, assuming the lower geometric correction factor or counting efficiency factor reported in earlier papers (Potten, 1977; Potten et al, 1988) or 3.5–4.5 cells per crypt using the factor from Table 1. Of these total apoptotic events, between two and three would be expected to occur within cell positions 3–7 in a whole crypt section (equivalent to about 2–4 susceptible stem cells).

The data as a whole suggest that, for the small intestine, there are a small number of apoptosis-susceptible cells per crypt (up to a maximum of six in total per crypt or four in the stem cell region per crypt) and that these are killed by a dose of 0.5–1.0 Gy. As crypts are not reproductively sterilized and destroyed by these doses, other stem cells must be more resistant (less prone to die by apoptosis). These are capable of regenerating the crypt and the apoptosis-susceptible population of stem cells per crypt given an appropriate time (Ijiri and Potten, 1984; Potten, 1995). These more resistant, apoptosis-insensitive cells probably represent the clonogenic compartment assayed using the crypt microcolony technique (Withers and Elkind, 1970; Potten and Hendry, 1985, 1995; Cai et al, 1997) and the p53-protein expressing cells seen at early times after irradiation (Merritt et al, 1994). Estimates of clonogenic cell numbers vary with radiation dose, i.e. with the levels of damage induced (Potten and Hendry, 1995), and these observations have been interpreted to suggest that the crypt may contain two tiers of clonogenic cells, as well as the ultimate steady-state stem cells, which are easily killed and die by apoptosis following small doses (Potten and Hendry, 1995; Potten et al, 1997).

Somewhat surprisingly, the plateauing effect with increasing dose was not observed with rat small intestine and mouse large intestine. In the rat small intestine, the yield of apoptosis increases progressively at all doses studied up to 10 Gy. In the murine large intestine the yield of apoptosis in all three regions studied increased progressively up to about 6 Gy, with the highest levels of apoptosis in the caecum, where the evidence of a plateau was weakest, and the lowest levels in the rectum. For doses lower than 1 Gy, fewer apoptoses were observed in all regions of the large bowel compared with the small intestine. The reasons for these differences between different regions of the bowel in the mouse and between mouse and rat small intestine remain obscure, but they are probably related to differences in the numbers and spatial distribution of susceptible (stem) cells within the crypt, or a more continuous spectrum of stem cell radiosensitivities (rather than discrete tiers) and/or differing activities of interacting apoptosis-related genes (*bcl-2*, *bax*, etc.) in these various regions.

### Cell position relationships

There are interesting spatial differences in the yield of apoptosis in the three regions of the large bowel of the mouse. For all three regions, the absolute yield of apoptosis following a dose of 1 Gy is lower than that seen in the small intestine. Somewhat contrary to earlier observations (Merritt et al, 1994), the distribution of apoptosis observed in the present study for the mid-colon, following radiation, shows a distinct cell positional characteristic as opposed to a broader distribution (Figure 2). The present data showed the highest levels of apoptosis near the bottom of the crypt. The caecal region of the large bowel has a spatial distribution similar to that seen in the small intestine, while the rectal region showed the highest levels of apoptosis at the base of the crypt with a more rapid fall off with increasing cell position than was observed in either the caecal or mid-colon region. If the apoptosis in the large bowel is similarly associated with the stem cell population, these observations suggest regional differences in the distribution of stem cells in the large bowel, as has been suggested by previous labelling studies (Kovacs and Potten, 1973; Sato and Ahnen, 1992), with stem cells at the very base of the crypt in the mid and rectal regions, but at higher positions in the caecum (perhaps distributed over cell positions 5–10). Current detailed cell proliferation and migration studies are under way to clarify further the location of the stem cell population in these three regions of the large bowel. Some of our earlier mid-colonic apoptotic observations may have been made on samples taken closer to the caecal region than those reported here. Sampling in the large bowel clearly needs to be carefully controlled.

Our earlier studies showed a clear inverse relationship between the levels of expression of the survival gene *bcl*-2 and the ease of inducing apoptosis with a dose or radiation (Merritt et al, 1995). There was little or no influence of *bcl*-2 on the ease of inducing apoptosis in the small intestine, where the Bcl-2 protein could not be detected, but the converse situation was observed in the large bowel where Bcl-2 was expressed. However, in both our earlier studies and recent investigations, the detection and demonstration of Bcl-2 protein using monoclonal antibodies on murine sections proved difficult and somewhat variable. The earlier observations were strongly supported by data obtained on apoptotic yield in *bcl-2* knockout mice (Merritt et al, 1995). In human colonic epithelium, the Bcl-2 expression pattern is stronger and much more reproducible. As the levels of Bcl-2 expression in mice may vary, the levels of apoptosis may similarly differ from time to time for unknown reasons. The data in Table 2 demonstrate that the average apoptotic index in mice can vary by up to a factor of 2.5. Similar levels of variability can be expected in knockout mice including the *bcl-2* nulls. This is currently seen in our *bcl*-2 null mice, which have considerably increased vigour. Our earlier data (Merritt et al, 1995) showed an increase in the peak levels of apoptosis (on cell positional plots) of up to tenfold for spontaneous apoptosis in the mid-colon, when the *bcl-2* nulls were compared with wild-type or conventional mice (BDF1), and an increase of about threefold in the peak level of apoptosis in the colon in nulls when analysed 3–6 h after 1 Gy. We are currently finding a similar degree of enhancement even though the absolute levels are almost a factor of 2 lower.

The implications for these observations on models of stem cell behaviour and function in the crypt, theories on the genetic and biochemical regulation of apoptosis, and hypotheses on the role of apoptosis in the carcinogenesis sequence are currently under further study.

### ACKNOWLEDGEMENTS

We are grateful to the Cancer Research Campaign for support and to Caroline Chadwick and Julie O'Shea for help with some of the experiments reported here.

### REFERENCES

Cai WB, Roberts SA and Potten CS (1997) The number of clonogenic cells in crypts in three regions of murine large intestine. *Int J Radiat Biol* **71**: 573–579

Duncan AMV, Ronen A and Blakey DH (1983) Diurnal variation in the response of gamma-ray-induced apoptosis in the mouse intestinal epithelium. *Cancer Lett* **21**: 163–166

Hendry JH and Potten CS (1982) Intestinal cell radiosensitivity: a comparison for cell death assayed by apoptosis or by a loss of clonogenicity. *Int J Radiat Biol* **42**: 621–628

Ijiri K and Potten CS (1983) Response of intestinal cells of differing topographical and hierarchical status to ten cytotoxic drugs and five sources of radiation. *Br J Cancer* **47**: 175–185

Ijiri K and Potten CS (1984) The re-establishment of hypersensitive cells in the crypts of irradiated mouse intestine. *Int J Radiat Biol* **46**: 609–623

Ijiri K and Potten CS (1987*a*) Further studies on the response of intestinal crypts cells of differing hierarchical status to eighteen different cytotoxic agents. *Br J Cancer* **55**: 113–123

Ijiri K and Potten CS (1987*b*) Cell death in cell hierarchies in adult mammalian tissues. In *Perspectives on Mammalian Cell Death*, Potten C S (ed.), pp. 326–356. Oxford University Press: Oxford

Ijiri K and Potten CS (1988) Circadian rhythms in the incidence of apoptotic cells and number of clonogenic cells in intestinal crypts after radiation using normal and reversed light conditions. *Int J Radiat Biol* **53**: 717–727

Ijiri K and Potten CS (1990). The circadian rhthym for the number and sensitivity of radiation-induced apoptosis in the crypts of mouse small intestine. *Int J Radiat Biol* **58**: 165–175

Kovacs L and Potten CS (1973) An estimation of proliferative population size in stomach, jejunum and colon of DBA-2 mice. *Cell Tissue Kinetics* **6**: 125–134

Li YQ, Fan CY, O'Connor PJ, Winton DJ and Potten CS (1992) Target cells for the cytotoxic effects of carcinogens in the murine small bowel. *Carcinogenesis* **13**: 361–368

Loeffler M and Potten CS (1997) Stem cells and cellular pedigrees – a conceptual introduction. In *Stem Cells*, Potten CS (ed.), pp. 1–27. Academic Press: London

Loeffler M, Bratke T, Paulus U, Li YQ and Potten CS (1997) Clonality and life cycles of intestinal crypts explained by a state development stochastic model of epithelial stem cell organisation. *J Theor Biol* **186**: 41–54

Merritt AJ, Potten CS, Kemp CJ, Hickman JA, Balmain A, Lane DP and Hall PA (1994) The role of *p53* in spontaneous and radiation-induced apoptosis in the gastrointestinal tract of normal and *p53*-deficient mice. *Cancer Res* **54**: 614–617

Merritt AJ, Potten CS, Watson AJM, Loh DY, Nakayama KI, Nakayama K and Hickman JA (1995) Differential expression of *bcl*-2 in intestinal epithelia. Correlation with attenuation of apoptosis in colonic crypts and the incidence of colonic neoplasia. *J Cell Sci* **108**: 2261–2271

Merritt AJ, Jones LS and Potten CS (1996) Apoptosis in murine intestinal crypts. In *Techniques in Apoptosis*, Cotter TG and Martin SJ (eds), pp. 269–299. Portland Press: London

Merritt AJ, Allen TD, Potten CS and Hickman JA (1997) Apoptosis in small intestinal epithelia from *p53* null mice: evidence for a delayed, *p53*-independent G2/M – associated cell death after irradiation. *Oncogene* **14**: 2759–2766

Paulus U, Potten CS and Loeffler M (1992) A model of the control of cellular regeneration in the intestinal crypt after perturbation based solely on local stem cell regulation. *Cell Proliferation* **25**: 559–578

Potten CS (1977) Extreme sensitivity of some intestinal crypt cells to X- and γ-radiation. *Nature* **269**: 518–521

Potten CS (1992) The significance of spontaneous and induced apoptosis in the gastrointestinal tract of mice. *Cancer Metastasis Rev* **11**: 179–195

Potten CS (1995) Structure, function and proliferative organisation of mammalian gut. In *Radiation and Gut*, Potten CS and Hendry JH (eds), pp. 1–31. Elsevier Science: The Netherlands

Potten CS (1996) What is an apoptotic index measuring? A commentary. *Br J Cancer* **74**: 1743–1748

Potten CS and Hendry JH (1985) The microcolony assay in mouse small intestine. In *Cell Clones: Manual of Mammalian Cell Techniques*, Potten CS and Hendry JH (eds), pp. 50–60. Churchill Livingstone: New York

Potten CS and Hendry JH (1995) Clonal regeneration studies. In *Radiation and Gut*, Potten CS and Hendry JH (eds), pp. 45–59. Elsevier Science: The Netherlands

Potten CS and Loeffler M (1990) Stem cells: attributes, cycles, spirals, pitfalls and uncertainties. Lessons for and from the crypt. *Development* **110**: 1001–1020

Potten CS, Al-Barwari SE, Hume WJ and Searle J (1977) Circadian rhythms of presumptive stem cells in three different epithelia of the mouse. *Cell Tissue Kinet* **10**: 557–568

Potten CS, Al-Barwari SE and Searle J (1978) Differential radiation response amongst proliferating epithelial cells. *Cell Tissue Kinet* **11**: 149–160

Potten CS, Roberts SA, Chwalinski S, Loeffler M and Paulus U (1988) Scoring mitotic activity in longitudinal sections of crypts of the small intestine. *Cell Tissue Kinet* **21**: 231–246

Potten CS, Li YQ, O'Connor PJ and Winton DJ (1992) A possible explanation for the differential cancer incidence in the intestine, based on distribution of the cytotoxic effects of carcinogens in the murine large bowel. *Carcinogenesis* **13**: 2305–2312

 © Cancer Research Campaign 1998

Potten CS, Booth C and Pritchard DM (1997) The intestinal epithelial stem cell: the mucosal governor. *Int J Exp Pathol* **78**: 219–243

Pritchard DM, Watson AJM, Potten CS, Jackman AL and Hickman JA (1997) Inhibition by uridine but not thymidine of *p53*-dependent intestinal apoptosis initiated by 5-fluorouracil: evidence for the involvement of RNA perturbation. *Proc Natl Acad Sci USA* **94**: 1795–1799

Qiu JM, Roberts SA and Potten CS (1994) Cell migration in the small and large bowel shows a strong circadian rhythm. *Epithelial Cell Biol* **3**: 137–148

Roberts SA, Hendry JH and Potten CS (1995) Deduction of the clonogen content of intestinal crypts: a direct comparison of two-dose and multiple-dose methodologies. *Radiat Res* **141**: 303–308

Sato M and Ahnen DJ (1992) Regional variability of colonocyte growth and differentiation in the rat. *Anat Record* **233**: 409–414

Tannock IF (1967) A comparison of the relative efficiencies of various metaphase arrest agents. *Exp Cell Res* **47**: 345

Watson AJM, Merritt AJ, Jones LS, Askew JN, Anderson E, Becciolini A, Balzi M, Potten CS and Hickman JA (1996) Evidence for reciprocity of *bcl-2* and *p53* expression in human colorectal adenomas and carcinomas. *Br J Cancer* **73**: 889–895

Weil MM, Stephens LC, Amos C, Ruifrok ACC and Mason KA (1996) Strain difference in jejunal crypt cell susceptibility to radiation-induced apoptosis. *Int J Radiat Biol* **70**: 579–585

Wimber DE, Quastler H, Stein OL and Wimber DL (1960) Analysis of tritium incorporated into individual cells by autoradiography of squash preparations. *J Biophys Biochem Cytol* **8**: 327–331

Withers HR and Elkind MM (1970) Microcolony survival assay for cells of mouse intestinal mucosa exposed to radiation. *Int J Radiat Biol* **17**: 261–267

© Cancer Research Campaign 1998